# Voluntary physical activity prevents insulin resistance in a tissue specific manner

**DOI:** 10.14814/phy2.12277

**Published:** 2015-02-23

**Authors:** Jessica L Sarvas, Jeffrey S Otis, Neelam Khaper, Simon J Lees

**Affiliations:** 1 Medical Sciences Division, Northern Ontario School of MedicineThunder Bay, Ontario, Canada; 2 Department of Biology, Lakehead UniversityThunder Bay, Ontario, Canada; 3 Department of Kinesiology and Health, Georgia State UniversityAtlanta, Georgia, Canada; 4 Health and Exercise Science, Colorado State UniversityFort Collins, Colorado, Canada

**Keywords:** Insulin sensitivity, liver, running, SOCS3

## Abstract

Physical inactivity and a sedentary lifestyle are risk factors for the development of type 2 diabetes. Here, we identified the effects 8 weeks of voluntary physical activity had on the prevention of insulin resistance in mouse skeletal muscles and liver (a hallmark of T2D). To do this, 8 week old C57BL/6J mice with (RUN) and without (SED) voluntary access to running wheels were fed a standard rodent chow ad libitum for 8 weeks. In the liver, there was a 2.5-fold increase in insulin stimulated Akt^SER^^473^ phosphorylation, and a threefold increase in insulin-stimulated (0.5 U/kg) GSK3β^SER^^9^ phosphorylation in RUN compared to SED mice. Although not induced in skeletal muscles, there was a twofold increase in SOCS3 expression in SED compared to RUN mice in the liver. There was no difference in the glucose tolerance test between groups. This study was the first to show differences in liver insulin sensitivity after 8 weeks of voluntary physical activity, and increased SOCS3 expression in the liver of sedentary mice compared to active mice. These findings demonstrate that even in young mice that would normally be considered healthy, the lack of physical activity leads to insulin resistance representing the initial pathogenesis of impaired glucose metabolism leading to type 2 diabetes.

## Introduction

Regular physical activity results in multiple physiological, biochemical, and metabolic changes that are critical for optimal health (Booth and Lees 2007). In contrast, the lack of physical activity has been linked to pathological changes in metabolic pathways, tissue and organ system dysfunction, and an increased risk of chronic disease such as type 2 diabetes (Lees and Booth 2004). Currently, approximately 347 million people suffer from diabetes; which will make it the seventh leading cause of death worldwide by 2030 (Mathers and Loncar 2006; Danaei et al. 2011). In addition to the impact on health, diabetes (particularly type 2 diabetes, or T2D) has a significant socioeconomic impact. For example, the American Diabetes Association recently estimated that the total costs of diagnosed diabetes have risen 41% over a 5 year period (2007–2012) to 245 billion dollars. Therefore, as the prevalence of T2D continues to increase, more viable and cost effective treatment strategies are essential. In addition to being more cost effective, studies have found that regular physical activity is more effective in the treatment of T2D than current pharmacological treatments (Knowler et al. 2002; Sharoff et al. 2010; Malin et al. 2012). In parallel, regular physical activity has been associated with reduced insulin resistance, a hallmark of T2D disease development and progression (Rector et al. 2011). Accordingly, understanding the impact of physical activity on the early molecular mechanisms associated with insulin responses (i.e., sensitivity or resistance) in tissues such as skeletal muscle and liver is crucial to effectively treat T2D.

Insulin is an anabolic hormone released by the β-cells in the pancreas to maintain glucose homeostasis within the body. The effects of insulin are complex with multiple signaling pathways involved in glucose regulation. For example, insulin leads to the phosphorylation and activation of Akt, which in turn, stimulates the translocation of GLUT-4 receptors to the plasma membrane and glucose uptake into skeletal muscle and liver tissues (Henriksen et al. 1990). In addition, glucose uptake can occur through insulin-independent, exercise-induced AMPK*α* phosphorylation following muscle contraction (Hayashi et al. 1998). Akt also phosphorylates its downstream target glycogen synthase kinase 3β (GSK3β). GSK3β inhibits glycogen synthase activity and prevents the conversion of glucose to glycogen (Sutherland and Cohen 1993; Cross et al. 1995; Taniguchi et al. 2006). Glycogen synthase inhibition is repressed when GSK3β is phosphorylated which allows for glycogen accumulation during periods of elevated blood glucose. However, key signaling factors have been implicated in the development of T2D. For example, suppressor of cytokine signaling 3 (SOCS3) proteins have been associated with insulin resistance by binding directly to specific sites on the insulin receptors (IRS-1/IRS-2) and targeting it for degradation (Ueki et al. 2004). Disrupting these early signaling events consequently causes decreased activity of downstream components in the insulin pathway, including PI3K and Akt phosphorylation, GLUT4 translocation, and glucose uptake (Senn et al. 2002, 2003; Yaspelkis et al. 2009; Zolotnik et al. 2012).

In addition to chronic diseases, physical inactivity is also a global pandemic, and a better understanding of the mechanisms linking physical inactivity to chronic diseases is necessary for prevention. Several studies have investigated the effects of physical activity on glucose metabolism and insulin signaling in rodent models (Krisan et al. 2004; Bernard et al. 2005; Kump and Booth 2005; Glynn et al. 2008). However, the majority of these studies have utilized supraphysiological doses of insulin in the investigation of insulin signaling. The purpose of the present study was three-fold. The first was to utilize a low physiological dose of insulin to determine the effects of voluntary physical activity on the prevention of insulin resistance in mice. The second was to detect early, tissue specific changes in insulin sensitivity between active and sedentary mice. Third, to determine if the regulation of SOCS expression is altered in sedentary mice without the influence of hyperphagia, high fat diet, or obesity.

It was hypothesized that voluntary physical activity would prevent the development of insulin resistance in the active mice.

## Research Design and Methods

### Animals

The C57BL/6J mice were purchased from Jackson Laboratory (Bar Harbour, ME, USA). Male mice (*n* = 8) were obtained at approximately 8 weeks of age, and studied after 1 week of acclimatization. Mice were housed under controlled temperature (18–20°C), humidity (40–70%), decibel level (<70 dB), and lighting (12 h of light; 12 h of dark) with free access to food and water. All animal experiments were performed in accordance with the institutional animal care committee guidelines at Lakehead University.

### Experimental protocol

The mice were fed a standard rodent chow ad libitum. Four mice had free access to running wheels. Over the 8 weeks of the study, running distances were recorded daily, and body weight was recorded weekly. Daily running distances were tracked using CatEye Velo 5 monitor, as we have previously described (Dekeyser et al. 2013; Sarvas et al. 2014).

### Glucose tolerance test

After 6 weeks, mice were morning fasted for 5 h prior to the glucose tolerance test (GTT). Baseline blood glucose levels were taken prior to a bolus intraperitoneal injection of glucose (1 g/kg). Blood samples were taken from the tail vein at baseline (0), and after 30, 60, 90, and 120 min. Glucose levels were measured with a hand held whole-blood glucose monitor (OneTouch Ultra2). The blood glucose response was quantified by calculating the area under the curve after the injection of glucose to describe the concentration of glucose in the blood 120 min after administration.

### Tissue collection

After 8 weeks, the mice were morning fasted for 5 h, then insulin was administered intraperitoneally at a dose of 0.5 U/kg. Ten minutes after the insulin injection, mice were anesthetized with isofluorane, and the hearts were removed. The liver, soleus, gastrocnemius/plantaris (combined) muscles were removed and immediately frozen in liquid nitrogen for further analysis. The gastrocnemius and plantaris muscles were combined due to their similar composition of type I and type II myosin heavy chain profiles. Serum was collected according to the Hepcidin Analysis serum and plasma collection protocol.

### Tissue lysis

Frozen muscle and liver tissues were homogenized in ice-cold lysis buffer (25 mmol/L Tris pH = 7.5, 150 mmol/L NaCl, 1 mmol/L EDTA, 1% Triton-X 100), and supplemented with phosphatase inhibitor cocktails 2 (Sigma, St. Louis, MO, P5726) and 3 (Sigma, P0044), and protease inhibitor cocktail (Sigma, P8340) at a dilution of 1:100. The tissues were homogenized using the Qiagen TissueLyser. The samples were centrifuged at 16 000 ***g*** for 10 min at 4°C, and then the supernatants were collected and stored at −80°C for further analysis. Protein assays were done using Bio-Rad DC Protein Assay (500-0116, Bio-Rad, Hercules, CA, USA) on all soleus muscle, gastrocnemius/plantaris muscle, and liver samples to determine the protein concentrations of these samples for further use in western blot analysis.

### Western blot analysis

A total of 45 *μ*g of protein was loaded and resolved by SDS-PAGE on 15% polyacrylamide gels, and transferred to nitrocellulose membranes. Immunoblotting was performed using the following primary antibodies: phosphorylated Akt^SER473^, GSK3β (Abcam, Cambridge, MA, USA); pan Akt, SOCS3, phosphorylated GSK3β^SER9^, phosphorylated AMPK*α*^THR172^, and total AMPK*α* (Cell Signaling Technology, Danvers, MA, USA). After incubation with either goat-anti rabbit (HRP) or goat-anti mouse (HRP) secondary antibody (Thermo Scientific Pierce, Rockford, IL, USA), the immunoreactive complexes were detected with enhanced chemiluminescence (ChemiDoc™ XRS, Bio-Rad, Hercules, CA, USA) and quantified by densitometry using ImageJ software.

### Enzyme-linked immunosorbent assay

To quantify the serum levels of insulin, Mercodia Insulin ELISA (Mercodia, Uppsala, Sweden) was used as per manufacturer's manual.

### Statistics

Data are presented as means ± SEM. Comparisons between groups were done using one-tailed Student's *t*-tests. Significance was accepted at *P *≤* *0.05.

## Results

### Body weight, running distance, and glucose tolerance test

**Figure 1:**
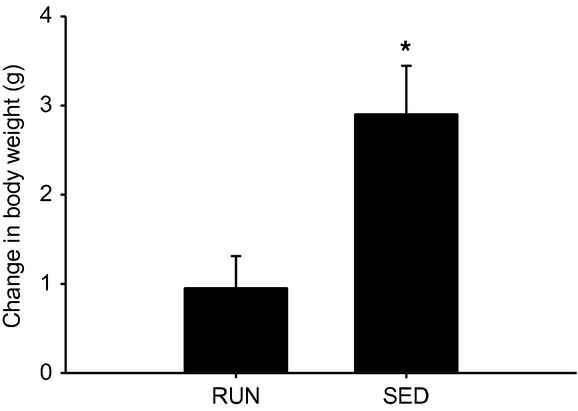
Sedentary mice showed increased weight gain compared to runners after 8 weeks. Change in body weight (grams) in mice with (RUN) and without (SED) voluntary access to running wheels for 8 weeks. *Significant differences (*P* ≤ 0.05) between groups. Data are presented as mean + SEM (*n* = 4 per group).

**Figure 2:**
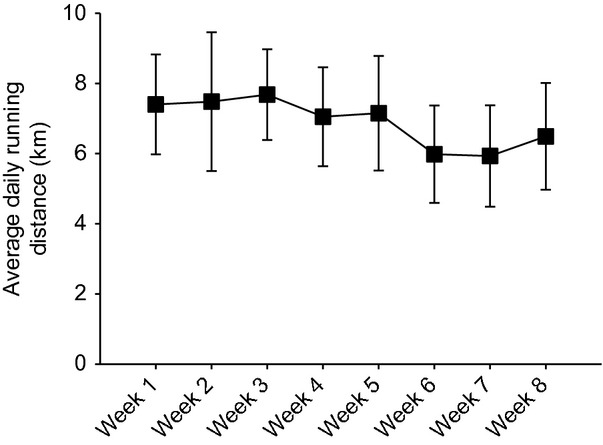
There were no differences in running distances during the 8 weeks. The average daily running distance (km) each week for mice with voluntary access to running wheels for 8 weeks. Data are presented as mean ± SEM (*n* = 4).

**Figure 3:**
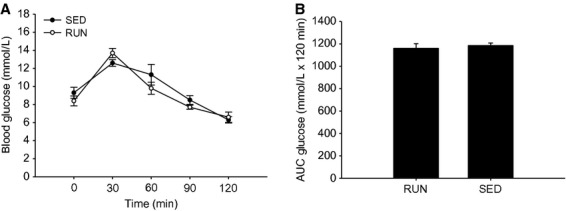
There were no differences in glucose tolerance after 6 weeks. (A) The mean blood glucose levels (mmol/L) following a bolus intraperitoneal injection of glucose in mice with (RUN) and without (SED) voluntary access to running wheels after 6 weeks. (B) The mean glucose area under the concentration-time curve (mmol/L × 120 min) for RUN and SED groups after 6 weeks. Data are presented as mean ± SEM (*n* = 4).

Initial body weights of mice in both running (RUN) and sedentary (SED) groups were taken and were not significantly different (27.8 ± 0.93 g and 26.1 ± 0.50 g, respectively), and then recorded weekly for 8 weeks. As expected, SED mice gained more weight compared to RUN mice (11.1% vs. 3.4%, *P* < 0.05) (Fig.1). Running distances remained constant over the 8 week duration of the study (Fig.2). After 6 weeks, there were no differences in fasting blood glucose levels between the groups (Fig.3A). In order to test glucose tolerance, mice were given a bolus intraperitoneal injection of glucose and circulating glucose concentration was measured every 30 min for 2 h. There were no differences in blood glucose observed at any time point of the GTT (Fig.3A). The calculated area under the curve for the GTT yielded no differences in blood glucose between the groups (Fig.3B).

### Phosphorylation and expression of signaling proteins

**Figure 4:**
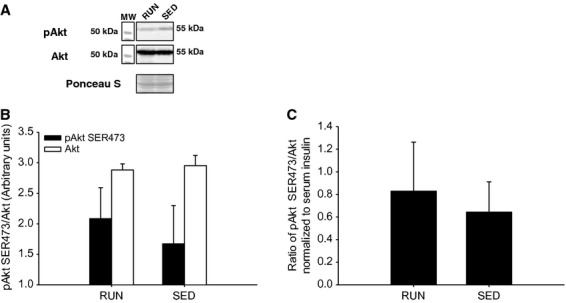
Insulin stimulated phosphorylation (SER473) of Akt in soleus muscle from mice with (RUN) and without (SED) voluntary access to running wheels for 8 weeks. (A) Representative western blot for pAkt and total Akt. (B) Black bars represent phosphorylated Akt protein expression (pAkt SER473). White bars represent total Akt protein expression (Akt). (C) Insulin stimulated phosphorylation of Akt represented and the ratio of pAkt to total Akt, then normalized to measured serum insulin concentration. Data are presented as mean + SEM, (*n* = 4 per group). Ponceau S stains are shown as markers of equal protein loading.

**Figure 5:**
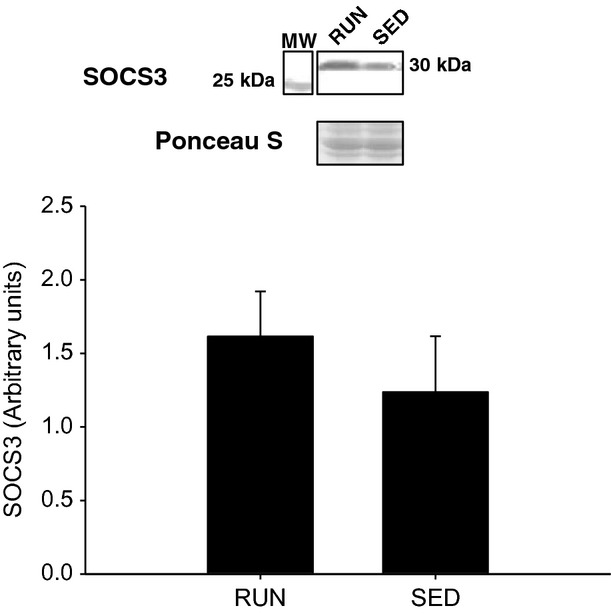
Soleus muscle SOCS3 protein expression from mice with (RUN) and without (SED) voluntary access to running wheels for 8 weeks. Data are presented as mean + SEM (*n* = 4 per group). Ponceau S stains are shown as markers of equal protein loading.

**Figure 6:**
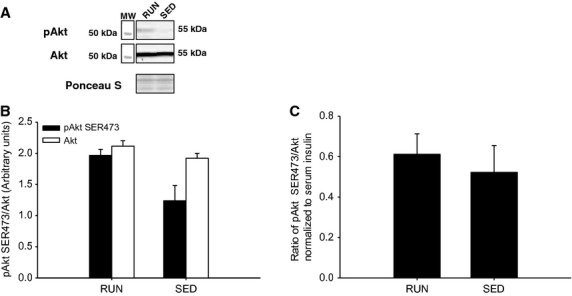
Insulin stimulated phosphorylation (SER473) of Akt in gastrocnemius/plantaris muscle from mice with (RUN) and without (SED) voluntary access to running wheels for 8 weeks. (A) Representative western blot for pAkt and total Akt. (B) Black bars represent phosphorylated Akt protein expression (pAkt SER473). White bars represent total Akt protein expression (Akt). (C) Insulin stimulated phosphorylation of Akt represented and the ratio of pAkt to total Akt, then normalized to measured serum insulin concentration. Data are presented as mean + SEM, (*n* = 4 per group). Ponceau S stains are shown as markers of equal protein loading.

After 8 weeks with or without voluntary physical activity, the phosphorylation and expression of signaling proteins associated with insulin sensitivity, insulin resistance, and physical activity were determined in soleus muscle, gastrocnemius/plantaris muscles, and liver**.** Serum insulin levels did not differ between groups (112.8 pmol/L ± 27.9 vs. 99.1 pmol/L ± 22.1, for RUN and SED, respectively) following a bolus intraperitoneal injection of insulin (0.5 U/kg) 10 min prior to tissue collection. In the soleus muscle, no differences in insulin stimulated Akt^SER473^ phosphorylation were found between groups (Fig.4A,B). In addition, there were no differences between groups when the ratio of phosphorylated Akt^SER473^ was normalized to circulating insulin (Fig.4C). As SOCS3 is known to regulate insulin sensitivity, protein expression was determined via western blot. There were no differences between groups in SOCS3 expression in the soleus muscle (Fig.5). Similar to the soleus muscle, there were no differences in insulin stimulated Akt^SER473^ phosphorylation between groups in the gastrocnemius/plantaris muscles (Fig.6A,B). When the ratio of phosphorylated Akt^SER473^ was normalized to circulating insulin in the gastrocnemius/plantaris muscles, no differences were observed between groups (Fig.4C). AMPK*α* phosphorylation (THR172) was not detected in either group in both the soleus and gastrocnemius/plantaris muscles.

**Figure 7:**
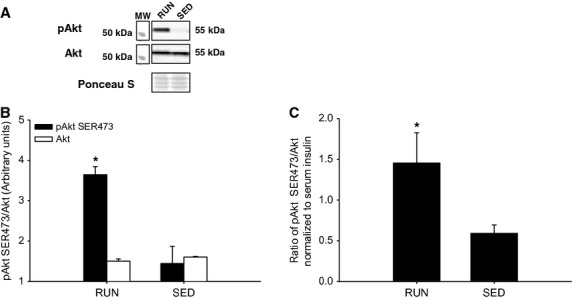
Insulin stimulated phosphorylation (SER473) of Akt in the liver from mice with (RUN) and without (SED) voluntary access to running wheels for 8 weeks. (A) Representative western blot for pAkt and total Akt. (B) Black bars represent phosphorylated Akt protein expression (pAkt SER473). White bars represent total Akt protein expression (Akt). (C) Insulin stimulated phosphorylation of Akt represented and the ratio of pAkt to total Akt, then normalized to measured serum insulin concentration. *Significant differences (*P* ≤ 0.05) between groups. Data are presented as mean + SEM, (*n* = 4 per group). Ponceau S stains are shown as markers of equal protein loading.

**Figure 8:**
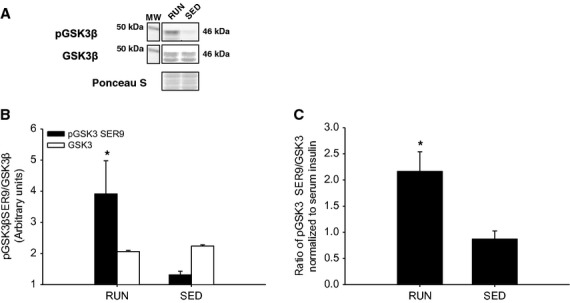
Insulin stimulated phosphorylation (SER9) of GSK3β in the liver from mice with (RUN) and without (SED) voluntary access to running wheels for 8 weeks. (A) Representative western blot for pGSK3β and total GSK3β. (B) Black bars represent phosphorylated GSK3β protein expression (GSK3β SER9). White bars represent total GSK3β protein expression (GSK3β). (C) Insulin stimulated phosphorylation of GSK3β represented and the ratio of pGSK3β to total GSK3β, then normalized to measured serum insulin concentration. *Significant differences (*P* ≤ 0.05) between groups. Data are presented as mean + SEM, (*n* = 4 per group). Ponceau S stains are shown as markers of equal protein loading.

**Figure 9:**
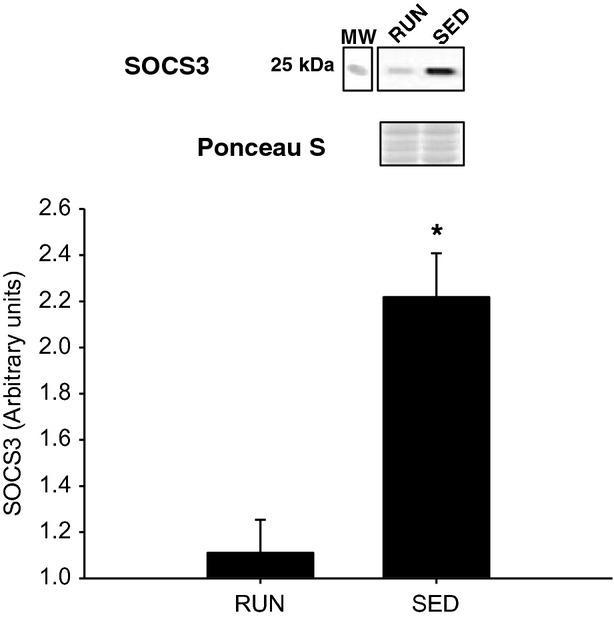
Liver SOCS3 protein expression from mice with (RUN) and without (SED) voluntary access to running wheels for 8 weeks. *Significant differences (*P* ≤ 0.05) between groups. Data are presented as mean + SEM (*n* = 4 per group). Ponceau S stains are shown as markers of equal protein loading.

**Figure 10:**
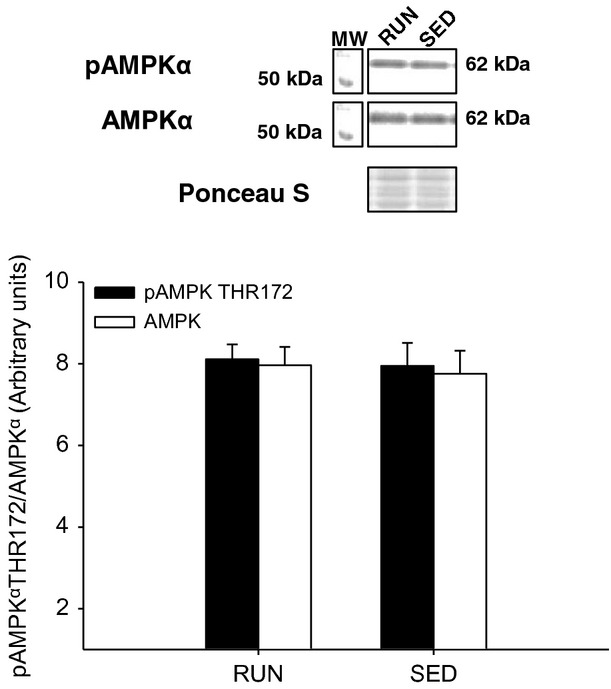
Phosphorylation (THR172) of AMPK*α* in the liver from mice with (RUN) and without (SED) voluntary access to running wheels for 8 weeks. Black bars represent phosphorylated AMPK*α* protein expression (pAMPK*α* THR172). White bars represent total AMPK*α* protein expression (AMPK*α*). Data are presented as mean + SEM (*n* = 4 per group). Ponceau S stains are shown as markers of equal protein loading.

In the liver, there was a 2.5-fold increase in insulin stimulated Akt^SER473^ phosphorylation in RUN compared to SED mice (*P* < 0.05) (Fig.7A,B). In addition, when the ratio of phosphorylated Akt^SER473^ was normalized to circulating insulin in the liver, the approximately 2.5-fold increase in the RUN group remained (*P* < 0.05) (Fig.7C). In order to investigate downstream insulin signaling in the liver, phosphorylation of GSK3β^SER9^ was measured. There was a threefold increase in insulin stimulated GSK3β^SER9^ phosphorylation in RUN compared to SED mice (*P* < 0.05) (Fig.8A,B). Similar to the phosphorylation of Akt^SER473^, when the ratio of phosphorylated GSK3β^SER9^ was normalized to circulating insulin in the liver, an approximately 2.5-fold increase in the RUN group remained (*P* < 0.05) (Fig.7C). In contrast with the skeletal muscles, there was a twofold increase in SOCS3 expression in SED compared to RUN mice in the liver (*P* < 0.05) (Fig.9). Unlike the skeletal muscles, AMPK*α*^THR172^ phosphorylation was detected in liver tissue. However, no differences in AMPK*α* phosphorylation were found between groups (Fig.10).

## Discussion

Regular physical activity can alleviate or protect against chronic disease by enhancing insulin sensitivity in peripheral tissue. However, less is known about early changes in insulin signaling that are a result of physical inactivity, yet precede the development of inactivity-related chronic diseases such as T2D (Ivy 1997; Hawley 2004; Krisan et al. 2004; Bradley et al. 2008; Hawley and Lessard 2008). Accordingly, the purpose of the present study was to determine the effects of voluntary physical activity on the prevention of insulin resistance in mouse skeletal muscle and liver tissues. Using a physiologically relevant dose of insulin, we have detected early signs of insulin resistance prior to the development of glucose intolerance and overt diabetes-like phenotypes. Here, we demonstrate two main findings that are novel and likely represent an early physiological adaptation to sedentary behavior. First, physical activity in what would normally be considered healthy animals resulted in 2–3 fold differences in insulin signaling at the liver. These results were tissue specific, in that skeletal muscle signaling was not found to be different. Second, differences in insulin signaling at the liver are observed in concert with significant decreases in SOCS3 expression. These findings represent a significant step forward in our understanding of the early adaptations that are a result of physical activity/inactivity in insulin mediated glucose metabolism.

There are significant differences in glucose metabolism and insulin action in mice compared to humans (McGuinness et al. 2009). Unlike humans, mice can lose up to 15% of their body mass overnight, and therefore, insulin action is enhanced during prolonged fasts (Andrikopoulos et al. 2008). To minimize the impact of an overnight fast (Ayala et al. 2010), the mice were fasted 5 h prior to insulin injection, and a physiological dose of insulin (0.5 U/kg) was chosen because it mimics postprandial insulin levels, and is commonly used for insulin tolerance test in both mice and humans (Andrikopoulos et al. 2008; Ayala et al. 2010). Together, the shortened fasting duration in combination with the 0.5 U/kg insulin bolus allowed the present study to investigate physiologically relevant differences in peripheral tissues in the presence or absence of physical activity. As a result, serum insulin levels at the time of tissue collection ranged from 99.1 to 112.8 pmol/L, which corresponds with previously reported postprandial insulin levels (60–150 pmol/L) in mice (Marguet et al. 2000; Chan et al. 2010; Tan et al. 2011; Yan et al. 2013).

Previous studies have utilized maximal or supraphysiological insulin doses in order to assess the effects of physical activity on glucose metabolism and the insulin-signaling pathway (Krisan et al. 2004; Bernard et al. 2005; Kump and Booth 2005; Glynn et al. 2008). Importantly, these nonphysiological doses of insulin create artificial responses not commonly observed in trained humans or rodents. For example, the effects of obesity and insulin resistance are not evident in insulin-stimulated glucose uptake and Akt phosphorylation (Krisan et al. 2004; Bernard et al. 2005; Kump and Booth 2005; Glynn et al. 2008). Furthermore, a maximal dose of insulin can also mask changes in insulin sensitivity leading to differences in glucose uptake and insulin signaling. This is demonstrated in a study by Kump and Booth (2005), which studied the effects of physical activity on insulin sensitivity using both maximal and submaximal insulin concentrations in vitro. When the maximal concentration was used, there was no difference in insulin-stimulated 2-deoxyglucose uptake in trained rat epitrochlearis muscles compared to sedentary controls. In sharp contrast, glucose uptake increased 37% when submaximal concentrations of insulin were used in their model (Kump and Booth 2005). Submaximal insulin also stimulated Akt^SER473^ phosphorylation. Together, these results suggest that changes in insulin-stimulated glucose uptake and insulin sensitivity are best detected using submaximal concentrations of insulin, and using maximal concentrations of insulin may underestimate or not detect changes to glucose uptake. In support of this notion, neither 12 weeks of aerobic training nor 12 weeks of resistance training altered Akt phosphorylation in rats when hind limbs were perfused with a maximal dose of insulin (Krisan et al. 2004; Bernard et al. 2005). Similar results have been shown in the liver, Farias et al. (2012) reported that insulin-stimulated Akt phosphorylation was not different between sedentary and exercise mice. However, the dose used in this study was supraphysiological, 1.2 nmol was injected directly into the circulation of mice, which have a blood volume of approximately 2 mL. This corresponds to approximately 600 nmol/L insulin in the circulation, whereas the serum levels in the present study ranged from 99.1 to 112.8 pmol/L.

In an effort to detect the effects of physical activity on changes in glucose tolerance, the GTT was conducted after 6 weeks in the present study. The mice were morning fasted for 5 h to mimic an overnight fast in humans due to metabolic differences, and administered a conservative dose (1 g/kg) of glucose prior to the test. The GTT revealed no differences in either fasting blood glucose or circulating glucose concentration at any time point during the test between the groups. Therefore, our findings of impaired insulin-mediated signaling in the liver precede changes to glucose tolerance. In most circumstances, insulin resistance is the earliest detectable defect in prediabetic animals and individuals. Several potential mediators of insulin resistance have been identified, including defects in insulin signaling in insulin sensitive tissues (Gerich 1999). Changes in insulin signaling in tissues such as skeletal muscle and liver generally precede changes in glucose tolerance. This occurs because increased insulin secretion can initially compensate for the developing insulin resistance. Once insulin secretion and insulin sensitivity deteriorate, whole body glucose tolerance becomes impaired (Gerich 1999). This was demonstrated by Seals et al. (1984) who found that the masters athletes, young athletes, and young untrained groups had similar glucose tolerance. However, the masters and young athletes exhibited a blunted plasma insulin response compared to the young untrained group (Seals et al. 1984). These findings support the present study, which highlighted significant differences in insulin signaling, but no differences in glucose tolerance between groups.

As mentioned above, previous studies have demonstrated that treadmill running (Bernard et al. 2005), swimming (Farias et al. 2012), and resistance training (Krisan et al. 2004) have not resulted in changes to insulin-mediated signaling in skeletal muscle. Our study was the first to investigate the in vivo response to a physiological insulin dose; however, even with this lower dose, we did not observe any differences to insulin-mediated phosphorylation of Akt^SER473^ in either the soleus or gastrocnemius/plantaris. While Kump and colleagues (Kump and Booth 2005) did report a difference to a submaximal insulin dose in the epitrochlearis muscle after voluntary wheel running, there are several differences in the experimental design that must be considered. First, our measured circulating insulin concentration was 99.1–112.8 pmol/L, whereas, Kump and Booth (2005) treated with nearly four times that level at 400 pmol/L. Second, our insulin treatment was in vivo, while Kump and Booth (2005) performed their study in vitro. Finally, Kump and Booth (2005) studied the epitrochlearis muscle, which is located superficially in the anterior forelimb of the rat and is primarily composed of myosin heavy chain (MHC) IIA and IIB (Nesher et al. 1980). In contrast, we analyzed posterior hindlimb muscles, specifically the mouse soleus, which is comprised of approximately 50% MHC type I fibers and gastrocnemius/plantaris complex, which is comprised of predominantly MHC type IIB, with varying type IIA and IIX (Henriksen et al. 1990; Bloemberg and Quadrilatero 2012).

SOCS3 and AMPK are known modulators of insulin signaling in both the liver and skeletal muscle (Sarvas et al. 2013) No differences in SOCS3 expression were found between RUN and SED groups in soleus muscle, and SOCS3 was not detected in either group in gastrocnemius/plantaris muscles. Physical activity can lead to increased AMPK phosphorylation, which has been linked to both increased insulin independent glucose uptake and enhanced insulin action in skeletal muscle (Hawley 2004; Hawley and Lessard 2008). Mice were morning fasted, and did not have access to running wheels for 5 h prior to tissue collection, which may have contributed to the lack of detection of AMPK*α* phosphorylation in the skeletal muscles. Physical activity and exercise training do not result in sustained increases in AMPK phosphorylation, and therefore, studies that have reported differences in skeletal muscle AMPK phosphorylation have minimized the length of time (2–40 min) between the cessation of physical activity and tissue collection (Musi et al. 2001; Park et al. 2002; Ruderman et al. 2006; Steinberg et al. 2010; O'Neill et al. 2013). Therefore, AMPK phosphorylation may not have been detected in skeletal muscle in the present study because it was no longer activated when the tissues were collected.

Physical activity and exercise training have also been shown to reduce or prevent insulin resistance in the liver in animal models of obesity or T2D (da Luz et al. 2011; Oliveira et al. 2011; Marinho et al. 2012; Yi et al. 2013; Sarvas et al. 2014). In parallel, physical activity may also result in increased AMPK, Akt, and GSK3β phosphorylation in the liver of rodents fed high fat diets (da Luz et al. 2011; Oliveira et al. 2011; Marinho et al. 2012; Yi et al. 2013). Additionally, voluntary wheel running resulted in reduced hepatic triglyceride levels, increased acetyl-coenzyme A carboxylase phosphorylation, and increased glucose uptake in rats fed a high fat diet (Rector et al. 2008, 2011; Chowdhury et al. 2013). In mice fed a standard chow diet, we show that insulin-stimulated Akt phosphorylation increased 2.5-fold, and subsequently, GSK3β phosphorylation increased threefold in the liver of RUN compared to SED mice. Although both AMPK and SOCS3 have been implicated in the control of hepatic glucose and lipid metabolism (Senn et al. 2003; Viollet et al. 2006), we were unable to detect significant differences in AMPK activation in livers from RUN mice compared to SED controls. Yet, SOCS3 expression was decreased by 50% in livers from RUN mice compared to SED controls and may reveal an important aspect to the development of insulin resistance in peripheral tissues. Further studies will be needed to confirm that this difference in SOCS3 expression due to physical activity is indeed an early step in the development of insulin resistance in humans. Previous studies investigating insulin stimulated Akt and GSK3β phosphorylation and SOCS3 expression in the liver have been limited to high fat diet and other models that rapidly induced obesity and chronic disease (Senn et al. 2002, 2003; Ueki et al. 2004, 2005). These findings are significant because regular physical activity prevented insulin resistance in mice in the absence of high fat diet.

Sedentary life style or physical inactivity can lead to increased systemic inflammation, obesity, and chronic disease. Increased systemic inflammation occurs due to the increased release and secretion of pro-inflammatory cytokines and mediators from the adipose tissue. These pro-inflammatory cytokines activate intracellular JAK-STAT signal transduction pathways. When these cytokines bind to their receptors on peripheral tissues, this leads to the phosphorylation and activation of JAK tyrosine kinases, and subsequently, STAT 3 proteins (Krebs and Hilton 2001). However, the duration and magnitude of cytokine signaling is tightly regulated. As phosphorylated STAT3 increases, this leads to increased expression of SOCS3 proteins, and SOCS3 provides negative feedback on the JAK-STAT pathway (Krebs and Hilton 2001). Due to the voluntary physical activity, which prevents the onset of inflammation, the RUN mice had decreased SOCS3 expression compared to the SED mice in the liver.

In conclusion, the present study demonstrated that physical activity modulates insulin sensitivity in the liver from otherwise healthy mice fed a normal diet. Furthermore, using a physiological dose of insulin highlighted the early changes in liver insulin signaling after only 8 weeks of voluntary physical activity. These collective findings suggest that voluntary physical activity can prevent the development of insulin resistance in a tissue-specific manner. Importantly, this study provides new insights into the use of physiological doses of insulin to detect early changes in glucose metabolism and insulin action in peripheral tissues. Together, our findings suggest that impaired insulin signaling and increased SOCS expression in the liver are impaired due to a lack of physical activity and represent early stages of metabolic dysfunction leading to type 2 diabetes. These findings emphasize the importance of regular physical activity and the prevention of inactivity-related diseases such as T2D.

## Conflict of Interest

No conflicts of interest, financial or otherwise, are declared by the authors.
